# Detecting variants with Metabolic Design, a new software tool to design probes for explorative functional DNA microarray development

**DOI:** 10.1186/1471-2105-11-478

**Published:** 2010-09-23

**Authors:** Sébastien Terrat, Eric Peyretaillade, Olivier Gonçalves, Eric Dugat-Bony, Fabrice Gravelat, Anne Moné, Corinne Biderre-Petit, Delphine Boucher, Julien Troquet, Pierre Peyret

**Affiliations:** 1 Clermont Université, Université d'Auvergne, Laboratoire: Microorganismes Génome et Environnement, BP 10448, F-63000 CLERMONT-FERRAND, France; 2 CNRS, UMR 6023, Laboratoire: Microorganismes Génome et Environnement, F-63173 AUBIERE, France; 3 Clermont Université, Université Blaise Pascal, Laboratoire: Microorganismes Génome et Environnement, BP 10448, F-63000 CLERMONT-FERRAND, France; 4 Biobasic Environnement, Biopôle Clermont-Limagne, 63360 Saint-Beauzire, France

## Abstract

**Background:**

Microorganisms display vast diversity, and each one has its own set of genes, cell components and metabolic reactions. To assess their huge unexploited metabolic potential in different ecosystems, we need high throughput tools, such as functional microarrays, that allow the simultaneous analysis of thousands of genes. However, most classical functional microarrays use specific probes that monitor only known sequences, and so fail to cover the full microbial gene diversity present in complex environments. We have thus developed an algorithm, implemented in the user-friendly program Metabolic Design, to design efficient explorative probes.

**Results:**

First we have validated our approach by studying eight enzymes involved in the degradation of polycyclic aromatic hydrocarbons from the model strain *Sphingomonas paucimobilis *sp. EPA505 using a designed microarray of 8,048 probes. As expected, microarray assays identified the targeted set of genes induced during biodegradation kinetics experiments with various pollutants. We have then confirmed the identity of these new genes by sequencing, and corroborated the quantitative discrimination of our microarray by quantitative real-time PCR. Finally, we have assessed metabolic capacities of microbial communities in soil contaminated with aromatic hydrocarbons. Results show that our probe design (sensitivity and explorative quality) can be used to study a complex environment efficiently.

**Conclusions:**

We successfully use our microarray to detect gene expression encoding enzymes involved in polycyclic aromatic hydrocarbon degradation for the model strain. In addition, DNA microarray experiments performed on soil polluted by organic pollutants without prior sequence assumptions demonstrate high specificity and sensitivity for gene detection. Metabolic Design is thus a powerful, efficient tool that can be used to design explorative probes and monitor metabolic pathways in complex environments, and it may also be used to study any group of genes. The Metabolic Design software is freely available from the authors and can be downloaded and modified under general public license.

## Background

Assessing the metabolic potential of microorganisms in variable ecosystems is a novel and stimulating challenge in biology. Microorganisms are present in all environmental habitats, even the most extreme, yet despite their ubiquity, we know relatively little about these communities. Microorganisms display vast diversity, each one having its own set of genes, cell components and metabolic reactions [1]. Thus 1 g of soil may contain up to 10^9 ^bacteria cells, which may represent between 1,000 and 10,000 different species [2,3]. Assuming 3,000 genes per single bacteria genome, there will thus be up to 3 × 10^12 ^genes mediating huge and various biological processes [3,4]. To overcome the limits of cultivation, several high throughput approaches have been developed to explore genetic contents, such as metagenomics or DNA microarrays [1,5,6]. Numerous random shotgun metagenomic projects have caused the publicly available sequence data to increase exponentially, giving us a basis to study complex ecosystems [1,5]. In some cases, these sequence data were used to identify different species in environmental or clinical samples with DNA microarrays [5]. Moreover, these data should improve our knowledge not only of genome organization and genome evolution but also of biological processes and biological activities. However, although such sequencing approaches can rapidly generate large amounts of data, they give only a snapshot of genetic information and can be laborious and costly when complex ecosystems are to be studied. Also, DNA sequencing is not informative on gene expression and regulation. Metatranscriptomic studies are promising, but several obstacles have to be crossed before they can be widely used [1,7]. Indeed, sequencing approaches highlight the difficulties of accurate functional annotation of unknown proteins without experimental data; unsupervised annotation of proteins by software pipelines suffers from very high error rates. Spurious functional assignments are usually caused by species homology-based transfer of information from existing database entries to new target sequences [8,9]. Such functional annotation errors are due to local similarities between the query and functionally annotated sequences. Hence, two protein sequences may have two different biological functions, but a same protein domain. This approach, based on homologous gene prediction, presents another major drawback: it can fail to identify novel enzymes that have the same function, but a different primary structure from known enzymes [10]. Today, the main sources for such protein sequence data are Swiss-Prot, TrEMBL and GenPept. This last should be considered as an equivalent to the Swiss-Prot/TrEMBL databases with a high level of redundancy in terms of protein sequences [11]. Unlike TrEMBL, the Swiss-Prot database contains curated datasets of high quality [12].

Another high throughput tool, functional DNA microarrays, can also be used for monitoring metabolic diversity of microbial populations in environmental samples. In a single experiment, thousands of genes can be simultaneously detected. Several studies already demonstrate the usefulness of functional DNA microarrays for exploring various ecosystems [13-15]. Hybridization of microarrays with mRNA targets permits low-cost, easy quantitative estimates of gene expression levels [16]. Monitoring environmental metabolic processes can be made more powerful, and so more useful, by designing explorative probes to ensure the detection of genes not already discovered and deposited in databases. However, microarray probe design software determines specific probes to monitor only known sequences [17]. Thus only a small fraction of genes encoding microbial enzymes can be studied with these probes. To solve this problem, degenerate probes need to be defined, as for PCR-based applications [18].

Probe design also has to allow for the constraints of cross-hybridization. Specificity is a measure of the inability of a probe to bind strongly to non-target sequences that may be present in a biological sample. This can be accomplished by avoiding probes with excessive sequence similarity to a non-target sequence that may be present during the hybridization [19,20]. These problems of cross-hybridization emphasize the need to take into account the fact that the studies are conducted on complex environments. As thermodynamic constraints are not yet completely understood [21], sequence similarity is currently the prime parameter used to check probe specificity. A previously reported and extensively cited work by Kane and coworkers [22] on 50-mer probes, shows that a probe must meet two conditions to be specific: (*i*) the oligonucleotide sequence must have no more than 75% similarity (among all sequences) with a non-targeted sequence present in the hybridization pool, and (*ii*) the oligonucleotide sequence must not include a stretch of identical sequence longer than 15 contiguous bases.

Here we describe a new algorithm, implemented in a user-friendly program, named Metabolic Design, which will generate efficient explorative probes using a simple convenient graphical interface. The practical utility of this approach was demonstrated by studying several genes encoding enzymes involved in the degradation of diverse polycyclic aromatic hydrocarbons from the model strain *Sphingomonas paucimobilis *sp. EPA505 (strain EPA505) and assessing metabolic capacities of microbial communities in a soil contaminated with aromatic hydrocarbons.

## Results

### The Metabolic Design software

**Figure 1:**
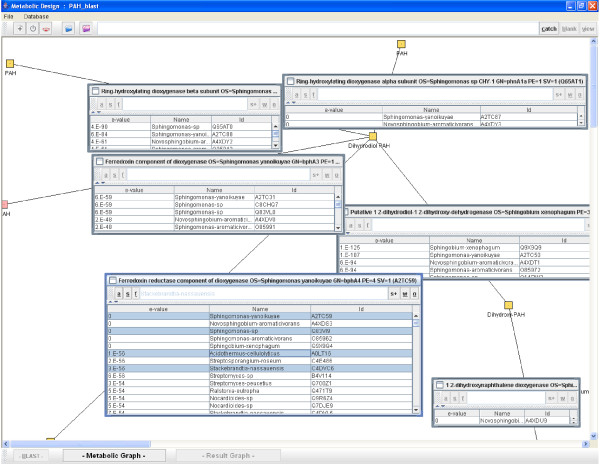
**Results window produced by Metabolic Design**. Each metabolite is represented by small yellow squares, called nodes, and enzymes as edges between nodes. Inner windows give the parsing of BLASTp results ordered by increasing Expected Value and obtained for each reference protein as query. For each extracted homologous protein sequence, data such as sequence in EMBL format (f button), sequence in FASTA format (s button), or split BLASTp alignment results (a button) are directly available through the inner window toolbar buttons. The w button, allows the execution of ClustalW alignment on pre-selected protein sequences. Such sequences can also be saved in a single file in FASTA format (s+ button), and/or used to launch the probe design module (o button). Additional functions have also been implemented. The user can automatically highlight potential metabolic capacities of a given organism (species name) subsequently using the 'catch' and 'view' buttons at the top of the window.

**Figure 2:**
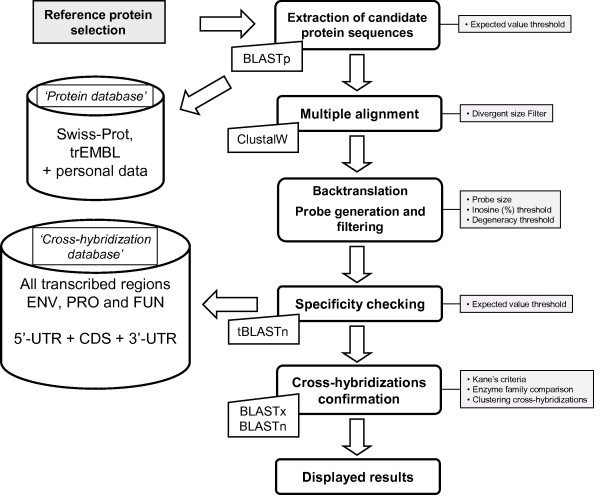
**Strategy to design explorative probes for functional microarrays used in Metabolic Design**. After extraction of potential candidate sequences by BLASTp using query reference protein to compare against concatenated Swiss-Prot and TrEMBL databases, a multiple alignment with selected protein sequences and the reference protein is performed. The next step is in two parts: (*i*) for each molecular site, amino acids are backtranslated, taking into account all genetic code redundancy to determine a degenerate nucleic consensus sequence, (*ii*) probes are then extracted from this consensus sequence, according to defined user parameters. The program then searches for all potential cross-hybridizations for each selected probe against the 'Cross-hybridization database' by tBLASTn. Kane's criteria are then checked for all positive results by BLASTn. If Kane's criteria are in agreement with a potential cross-hybridization, the program also checks whether it is a potential member of the targeted enzyme family using a BLASTx comparison against the reference protein. Cross-hybridization results are then clustered by BLASTn, stored and visualized in an output file.

Our aim is to build a graphical display of given biological processes and perform exhaustive sequence mining of all available protein sequences for each biological step studied. The graphical user interface (GUI) allows for example the graphical reconstruction of tailor-made metabolic pathways, with metabolites and enzymes represented respectively with nodes and edges (Figure 1). Using appropriate keywords, correctly annotated protein sequences are extracted from a curated database (by default Swiss-Prot potentially enriched with personal data) for each edge of the graph. The user can freely select the most suitable protein as a reference sequence query. This sequence is then used to carry out exhaustive mining of similar proteins from public and/or personal databases. The strategy of probe design using Metabolic Design software is described in Figure 2 and detailed in Methods under 'Software implementation'.

In our study, reference sequences are extracted from the highly curated database Swiss-Prot formatted for the application to ensure efficient mining. In addition, the reference database is enriched with TrEMBL protein sequences biologically validated when a non-studied orthologous protein sequence was found in the Swiss-Prot database. For every listed protein, data are extracted using a homologous approach with a BLASTp program against concatenated Swiss-Prot and TrEMBL databases. Thus the selection of candidate protein sequences based on similarity criteria bypasses functional annotation errors. Extracted sequences are then automatically filtered and displayed in graphical edges for each studied enzyme. The results are finally organized according to increasing expected value, or organism origin, and miscellaneous functions are also implemented in the toolbar to facilitate additional data extraction and visualization (Figure 1).

**Figure 3:**
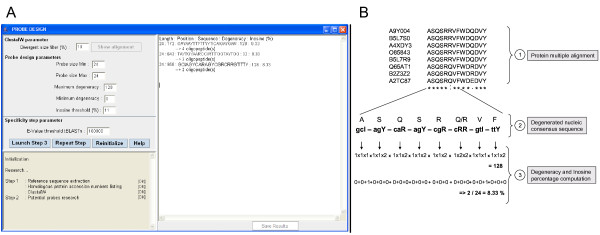
**Probe determination with Metabolic Design**. A: The inner window of Metabolic Design showing results for designing probes with Metabolic Design and parameters (such as probe size, degeneracy or inosine composition) defined by the user. The user's chosen parameters are visible on the left, and potential probes are listed on the right. The program also displays all potential peptide combinations for each degenerate probe (named as oligopeptides) with probe listing. B: Example of probe design approach, degeneracy calculation and inosine percentage determination for the third probe in the inner window. Note that inosine residues are not taken into account for the degeneracy calculation step.

This multiple alignment is then used to design specific explorative oligonucleotide probes targeting studied proteins (Figure 3A), using the following procedure. To reduce insertion-deletion (indel) regions in multiple alignments, a first filtering step is carried out to exclude sequences with high size divergence compared with the reference query. A degenerate nucleic consensus sequence based on the IUPAC (International Union of Pure and Applied Chemistry) nomenclature is defined from the protein multiple alignment using the backtranslation approach [23]. For each molecular site, potential amino acids are backtranslated taking into account all genetic code redundancy (Figure 3B). Probes are then extracted from this consensus sequence, according to three defined user parameters: probe size, degeneracy and inosine composition thresholds. Along the consensus sequence, the algorithm extracts all probes by incrementing the constant defined probe size in a window. All probes with degeneracy and inosine composition under the set thresholds are then listed in an inner window in the GUI. Thus the user can select all or some pre-selected potential probes for specificity testing. To reduce computing time, during this test the algorithm generates all peptide combinations for each degenerate probe. Thus the degeneracy code redundancy is bypassed and the number of comparisons is greatly reduced. This test is carried out using tBLASTn against the 'Cross-hybridization database' using Kane's algorithm criteria [22]. Indeed, those parameters are used to check all positive results by comparison at the nucleotide level with BLASTn. If those criteria are in agreement with a potential cross-hybridization this may also reflect hybridization with a member of the targeted enzyme family. To avoid this bias, the algorithm extracts the complete sequence of the gene harboring the potential cross-hybridization region and compares it with the reference protein using the BLASTx program. Finally, a file containing all potential cross-hybridizations for every candidate probe is automatically clustered, created and displayed.

### Data mining and probe selection for microarray experiments using Metabolic Design

To validate our probe design strategy, we focus on metabolic pathways involved in the biodegradation of polycyclic aromatic hydrocarbons (PAHs). PAHs are a class of fused-ring aromatic compounds that are ubiquitous environmental pollutants known to be toxic, mutagenic and/or carcinogenic. Many researchers have therefore focused on the biodegradation of these pollutants by microorganisms, especially bacteria. Several enzymes involved in these critical biodegradation steps have been characterized and their sequences deposited in databases [24-28].

In this study, we choose to target eight genes (*phnA1a*, *phnA2a*, *bphC*, *bphA3*, *ahdA1c*, *ahdA2c*, *ahdA4 *and *bphB*) (Table 1) known to be involved in the degradation of several PAHs (such as phenanthrene (PHE) and fluoranthene (FLA)). Using our defined data mining strategy, we first construct the metabolic pathway with respective substrates and products of each metabolic step. Secondly, for each of these metabolic steps, one reference enzyme is extracted from our curated database (Swiss-Prot and trEMBL validated data). Homologous proteins are then retrieved from complete databases (Swiss-Prot and TrEMBL). Based on defined expected threshold values, different sequences are selected (Table 1) and multiple alignments are then performed to ensure probe design step.

**Table 1:** Reference enzyme information.

	REFERENCE PROTEIN				BLAST THRESHOLD AND SEQUENCES USED	
**Gene**	**Enzyme**	**Organism**	**Accession Number**	**Reference**	**BLASTp e-value**	**Chosen enzymes for the probe design**

*phnA1a*	Putative alpha subunit of ring-hydroxylating dioxygenase	*Sphingomonas *sp. CHY-1	Q65AT1	[24]	1e-40	B2Z3Z2, A2TC87, A4XDY3, 085843, A9Y004, B5L7S0, B5L7R9, Q1HCP6, Q7WUA0

*phnA2a*	Putative beta subunit of ring-hydroxylating dioxygenase	*Sphingomonas *sp. CHY-1	Q65AT0	[24]	1e-30	A2TC88, A4XDY2, 085842, B5L7R8

*ahdA1c*	Putative large subunit of oxygenase	*Sphingomonas *sp. P2	Q83VL2	[27,32]	1e-40	A2TC29, A9XZZ2, Q65AS5

*ahdA2c*	Putative small subunit of oxygenase	*Sphingomonas *sp. P2	Q83VL1	[27,32]	1e-30	A9XZZ3, Q65AS6, A4XDV1, 085992, A2TC30, Q9Z4T6

*bphB*	Putative 1,2-dihydrodiol-l,2-dihydroxy-dehydrogenase	*Sphingobium xenophagum*	Q9X9Q9	[25]	1e-40	Q14RW3, 085972

*bphC*	Putative biphenyl-2,3-diol 1,2-dioxygenase	*Sphingobium xenophagum*	P74836	[25]	1e-40	PI 1122, Q6LCU9, Q7DG81, A4XDU9, 085990, A9XZZ5, Q65AS8, Q9KWI2

*bphA3*	Putative ferredoxin component of dioxygenase	*Sphingomonas yanoikuyae*	A2TC31	[24,32]	1e-20	034128, Q65AS7, A9XZZ4, A4XDV0, 085991, Q83VL0

*ahdA4*	Putative ferredoxin reductase component of dioxygenase	*Sphingomonas yanoikuyae*	A2TC59	[24,32]	1e-40	Q83VI9, A4XDS3, 085962

Organism name and source, accession number and bibliographic reference for each reference protein. BLASTp expected threshold values used and selected sequences for multiple alignments for the probe design are given.

To improve our probe design, we have applied two different strategies, using the same multiple alignments. In these strategies, we set the probe length at 24-mer, representing the best compromise between probe specificity and sensitivity criteria [29]. In the first strategy, (degeneracy threshold: 129, inosine threshold: 25%), we have determined a first set of probes for each targeted enzyme. However, owing to high percentages of inosine, these probes generally show a high degree of total degeneracy. Indeed, like the inosine residues are not taken into account for the degeneracy threshold determination, probes may present a maximum total degeneracy of 528,384. To reduce the number of potential specific probes derived from each degenerate defined probe, a second strategy with more stringent parameters is applied (degeneracy threshold: 258, inosine threshold: 9%, maximum total degeneracy of 4,128). Using these parameters, we have found another set of probes for each targeted enzyme. We then choose among the two probe sets obtained with these two strategies, the best probes based on several sequentially evaluated criteria: (1) the total number of potential cross-hybridizations to decrease possibilities of non-homologous hybridizations, (2) the probe total degeneracy (including inosine composition) to restrict the number of specific probes in the microarray, and (3) the position of each probe in the reference sequence to target different regions for each enzyme. Also, to reduce the number of specific probes synthesized on the microarray, the last nucleotide of each probe (generally a degenerate base or an inosine due to degeneracy of the genetic code) is also manually eliminated.

Thus by these strategies, two degenerate probes targeting two different regions are selected per targeted gene (Table 2). Based on these sixteen 23-mer degenerate probes, we finally obtain 8,048 specific probes.

**Table 2:** Selected probe information.

Targeted Gene	Probe name	Sequence	Number of unique DNA sequences used for the probe design	Number of specific probes	Positions on the reference gene sequence
*phnA1a*	phnA1a_MD_A	GTITGYAAYTAYCAYGGITGGGT	5	256	294 - 316
	phnA1a_MD_B	CAYGARATHGARGTITGGACITA	4	384	957 - 979

*phnA2a*	phnA2a_MD_A	GARGAYATHCAYTAYTGGATGCC	2	48	123 - 145
	phnA2a_MD_B	GGICARGTITGGATGGARGAYCC	3	128	261 - 284

*ahdA1c*	ahdA1c_MD_A	GARTGYGTITAYCAYCARTGGGC	3	128	318 - 340
	ahdA1c_MD_B	GAYGCIGCIGAYAARCARGCITA	2	1024	771 - 793

*ahdA2c*	ahdA2c_MD_A	GAYGAYMGIYTIGARGARTGGCC	3	1024	081 - 103
	ahdA2c_MD_B	ATHGAYACIATGATGGTIMGICC	3	768	459 - 481

*bphB*	bphB_MD_A	AAYGTIGGIATHTGGGAYTWYAT	3	768	261 - 283
	bphB_MD_B	AAYBTIAARGGITAYTTYTTYGG	3	384	348 - 370

*bphC*	bphC_MD_A	CCITAYTTYATGCAYTGYAAYGA	5	128	558 - 580
	bphC_MD_B	TGGYTITGGGARTTYGGITGGGG	4	128	777 - 799

*bphA3*	bphA3_MD_A	ATHATHGARTGYCCITTYCAYGG	2	576	180 - 202
	bphA3_MD_B	ATHGAIGAYGGITGGGTITGYAT	3	768	279 - 302

*ahdA4*	ahdA4_MD_A	GCIAAYGTICCIGAYAAYTTYTT	2	1024	159 - 181
	ahdA4_MD_B	CARGARACITAYCARAAYGCIGC	2	512	867 - 889

Total number of specific probes from the probe degenerate sequence and relative positions on the reference gene sequence for each targeted gene are described._Numbers of unique DNA sequences, coding for studied enzymes are also given to highlight that our probes target known genes but also unknown ones. Nomenclature: **M**: A and C; **R**: A and G; **W**: A and T; **S**: G and C; **Y**: C and T; **H**: A, C and T; **D**: A, G and T; **B**: G, T and C; **I**: A, C, G and T.

### Explorative probe validation

Strain EPA505 is known to utilize PHE and FLA as sole sources of carbon and energy for growth [30]. However, for this strain, the enzymes involved in the catabolism of PHE and FLA have not been fully characterized. Only gene fragments for the ferredoxin component of dioxygenase (*pbhB *equivalent to *bphA3*) and for the 1,2-dihydroxy-biphenyl-2,3-diol 1,2-dioxygenase (*pbhA *equivalent to *bphC*) are available in public databases [31] for the studied enzymes. This strain is thus an excellent model to validate our approach, as we could work with no prior assumptions using explorative probes to ensure the detection of unidentified genes.

With this aim, growth kinetics experiments with PHE, FLA and a mix of both pollutants as sole carbon and energy source are carried out to evaluate the targeted gene expression. As expected, for the eight genes studied, we have detected positive hybridizations (SNR' > 3) on the DNA microarray using mRNA as targets extracted after 3 h of culture (Table 3). Surprisingly, we do not observe positive signals with the probes targeting one region of the *phnA2a *gene. However, one probe targeting the second region of this gene allow the detection of strong hybridization signals (SNR' = 22.64 

 ±

  3.21) indicating a potentially high level of gene expression induced by PAHs. Additionally, control experiments with glucose as sole carbon and energy source do not give positive hybridizations for most of the targeted genes (Table 3). The SNR' value indicating positive hybridization is close to the threshold reflecting a low gene expression. These results suggest that all the studied genes can be induced in response to the mix of PAH exposure. The same results are obtained for growth kinetics with one PAH (PHE or FLA) as sole carbon and energy source. The same specific probes give the highest SNR' for the eight targeted genes, but with different levels of induction. For example, for the same specific probe (named bphA3_MD_B_0333) targeting the region B of the gene *bphA3 *in all PAH-cultures we find: 9.79 

 ±

  1.39 with a mixture of two pollutants, 20.00 

 ±

  5.84 with PHE alone, 7.50 

 ±

  2.03 with FLA alone and no positive signal with glucose. We note that the number of probes giving a positive signal is low for targeted genes (between 1 for *phnA2a *and 5 for *bphB *after 3 h of culture with the mix of PAHs) reflecting variable levels of similarity between targets and probes deduced from variably degenerate regions.

**Table 3:** Results obtained with designed probes for a mixture of phenanthrene and fluoranthene.

Gene name	*phnA1a*		*phnA2a*		*ahdA1c*		*ahdA2c*		*bphB*		*bphC*		*bphA3*		*ahdA4*	
**Targeted region**	**A**	**B**	**A**	**B**	**A**	**B**	**A**	**B**	**A**	**B**	**A**	**B**	**A**	**B**	**A**	**B**

**Total number of ** **specific probes**	256	384	48	128	128	1024	1024	768	768	384	128	128	576	768	1024	512

**Number of specific ** **probes giving a positive ** **signal (SNR' > 3)**	1	2	0	1	3	1	2	1	4	1	1	1	3	1	0	0

**Highest median SNR' ** **obtained for each ** **targeted region**	18.32 ± 3.64	6.62 ± 0.31	X	22.64 ± 3.21	8.61 ± 1.59	9.93 ± 1.32	8.92 ± 1.52	16.26 ± 2.45	5.79 ± 1.73	4.09 ± 0.66	4.47 ± 0.30	4.54 ± 0.81	36.87 ± 7.83	9.79 ± 1.39	X	X

**Specific probe for ** **EPA505 gene giving ** **highest median SNR'**	Yes	No	No	Yes	No	Yes	Yes	Yes	Yes	No	Yes	No	Yes	Yes	No	No

**For comparison, total ** **number of specific probes ** **giving a positive signal ** **with glucose**	0	0	0	0	0	0	1	0	0	0	0	0	0	0	0	0

For each degenerate probe defined targeting two different regions (A and B) of genes (*phnA1a, phnA2a, ahdA1c, ahdA2c, bphB, bphC, bphA3 *and *ahdA4*), total number of specific probes stemming from the degenerate sequence, total number of specific probes giving a 'positive' signal (with a SNR' > 3), highest median SNR' visualized for each targeted region of each gene and whether the probe specific to the strain EPA505 gene gives this highest signal median SNR'.

Based on these results, we can also predict the most likely gene sequence of the targets interacting with probes. Among the positive probes, one shows a strong signal (e.g. one targeting *bphA3 *with a median SNR' = 36.87 

 ±

  7.83) compared with the others targeting the same region. We hypothesize that the strongest SNR' probe perfectly matched, or is the closest sequence to targeted genes. Using sequences of *bphA3 *and *bphC *genes available in databases [EMBL: AF259397 and AF259398], we demonstrate that only two probes among the four have identical sequences with *bphC *and *bphA3 *genes. These data do not confirm the efficiency of our approach, and so to validate our first observations, we decide to isolate and characterize these genes and the others by a combination of amplification, cloning and sequencing strategies. Four gene clusters of 4.47 kb, 2.13 kb, 1.20 kb, and 0.32 kb, respectively [EMBL: FM882255, FM882254, FM882253 and FN552592] are thereby obtained. The complete nucleotide sequence of the 4.47 kb contig [EMBL: FM882255] shows six putative non-overlapping open reading frames (ORFs). Among these, four are targeted with our microarray probes. The first encodes a polypeptide 98% similar to a putative biphenyl-2,3-diol 1,2-dioxygenase known to degrade various dihydroxy-PAHs, and named BphC [EMBL: BAC65429]. The second encodes a polypeptide 90% similar to a putative ferredoxin component of dioxygenase, named BphA3 [EMBL: BAC65428], involved in various steps of the process of PAH degradation for the electron transfer from reductase to dioxygenase complex [26]. Interestingly, these two ORFs are highly similar to available sequences for strain EPA505 [31], but a comparison with our sequences reveals some mismatches. The two last genes encode two polypeptides respectively 88% and 95% similar to AhdA2c [EMBL: BAC65427] and AhdA1c [EMBL: BAC65426], two components of a terminal oxygenase involved in the monooxygenation of salicylate, a metabolic intermediate of PHE, to catechol [32,33]. Two genes identified on the 2.13 kb contig (FM882254) encode polypeptides of 455 and 175 residues. These polypeptides resemble in length and sequence the alpha (99% sequence identity) and beta (100% sequence identity) subunits [EMBL: CAG17576 and CAG17577] of the ring-hydroxylating dioxygenase (*phnA1a *and *phn2a *respectively) of *Sphingomonas *sp. CHY-1, involved in the conversion of several PAHs into their corresponding dihydrodiols [28,34]. The third contig of 1.20 kb (FM882253) encompasses a single partial ORF encoding a polypeptide displaying 95% similarity with the ferredoxin reductase component of a dioxygenase, named AhdA4 [EMBL: BAC65450] of *Sphingobium *sp. P2 and involved in the electron transfer in association with BphA3 [35]. The last contig of 0.32 kb [EMBL: FN552592] encodes a partial 107 amino acid sequence 97% similar to a 1,2-dihydrodiol-1,2-dihydroxy-dehydrogenase named BphB [EMBL: ABM79802] of *Sphingobium yanoikuyae *B1.

Comparison of these gene sequences with the microarray probes shows that our design strategy is efficient to detect, with no prior sequence assumptions, targeted genes from complete metabolic pathways. As expected, for each gene, different probes give positive signals in agreement with the gene sequence composition. Furthermore, among the thirteen probes (targeting both regions of the eight genes) giving the highest signals, nine probes perfectly match strain EPA505 targeted gene regions (Table 3). Thus the two regions (A and B) selected for *bphA3 *and *ahdA2c *genes probe designs allow the specific identification of these genes. For the genes *phnA1a*, *phnA2a*, *ahdA1c*, *bphB *and *bphC*, only one region can be considered specific for the identification of the genes. Finally, for *ahdA4 *gene, as no probes give positive signals, we can then hypothesize that *ahdA4 *is not expressed or is weakly expressed (under the detection threshold) in our culture conditions. We can also postulate that absence of signal might reflect a low sensitivity of these selected probes targeting *ahdA4*.

To conclude, these results confirm that our design strategy is useful and efficient for the targeted genes studied. These data also show that it is essential to select at least two specific regions for each studied gene that should be experimentally validated to ensure accurate identification. Nevertheless, a majority of selected regions is useful for the design of efficient probes that perfectly hybridize with their targets and show the strongest signal on the microarray.

### Gene expression analysis with microarray and quantitative real-time PCR experiments

As described previously, the applied design strategy lets us to detect targeted genes from the studied metabolic pathway without prior assumptions. It is thus of interest to test whether our DNA microarray is able to evaluate mRNA levels semi-quantitatively during biodegradation kinetics with PHE, FLA and a mixture of the two pollutants as sole carbon and energy source. A control experiment with glucose as sole carbon and energy source is also conducted. For these four conditions, total RNAs are extracted from pure cultures of strain EPA505 at different times of the kinetics (0, 3, 6, 10 and 21 h). According to the explorative probe validation conclusions (see previous section), only the most efficient probes targeting each of the eight genes in response to pollutant exposure are considered. In addition, to evaluate the gene expression level, a quantitative reverse transcription PCR approach is also developed for the selected genes during the same times of the kinetics.

Transcript hybridizations obtained with only glucose-amended cultures give no positive probe signals (SNR' > 3) for the different times of the kinetics studied as shown in Additional file 1. Under PHE-growth conditions, specific probes give positive signals (SNR' > 3) after 3 h of growth for all the studied genes (Additional file 1). Detected signals largely decrease at 6 h of culture to reach SNR' values under the set threshold. Same SNR' values, in agreement with absence or low abundance of targeted mRNA, are also obtained after 10 h and 21 h of culture (Additional file 1). With FLA as carbon source, except for *ahdA1c*, *bphC *and *bphB *probes, positive SNR' values are also obtained with specific probes after 3 h of growth. After 6 h of culture with FLA, no positive probe signal (SNR' > 3) is detected, as in glucose-growth conditions (Additional file 1). Surprisingly, a positive signal for the specific probe targeting *bphB *is detected after 6 h of culture (SNR' = 3.43 

 ±

  0.70) with FLA, but not after 3 h of culture. Finally, with a mixture of the two pollutants, high positive signals are detected, except for the *ahdA4 *gene, under the SNR' threshold and for *bphC *and *bphB*, just above the SNR' threshold, after 3 h of culture (Additional file 1). After a large decrease in SNR' values after 6 h of culture, positive signals for most of the probes are visualized after 10 h of culture, indicating a new gene expression induction. Finally, at 21 h of culture, the detected signals have the same SNR' values as those obtained with glucose. Gene expression results obtained with microarray assays show an up-regulation of all the studied genes with different mRNA levels according to PAH exposure (Additional file 1). For *ahdA4*, no positive signals are detected except with PHE after 3 h of culture with a SNR' close to the threshold (SNR' = 3.19 

 ±

  0.40).

At the same time, a quantitative reverse transcription PCR based approach is used to precisely describe the gene expression during the growth kinetics. Results show the same expression profiles as those observed with DNA microarray experiments (Additional file 1). Low mRNA levels are detected during growth on glucose, indicating a very low basal gene expression in the absence of PAH substrates. With PHE or FLA as sole carbon and energy source, a high level of targeted mRNA is detected after 3 h of growth. However, a higher mRNA level is detected with PHE exposure. For these two cultures, after 10 h of culture, gene transcript number decreases to reach mRNA levels close to or below the control copy number detected in glucose-grown cells, as with results visualized with the DNA microarrays. With a mixture of the two pollutants, the same expression profile is detected with the quantitative reverse transcription PCR approach and with the DNA microarrays. High mRNA levels are measured after 3 h of culture, and besides a large decrease after 6 h of culture, another mRNA up-regulation is detected at 10 h of culture for the studied genes. Finally, mRNA levels decrease to reach transcript levels close to growth experiments performed with glucose. In conclusion, similar expression profiles are obtained for *phnA1a, phnA2a*, *ahdA1c*, *ahdA2c*, *bphB*, *bphC *and *bphA3 *with DNA microarray and quantitative reverse transcription PCR approaches, demonstrating the efficiency of probes designed using Metabolic Design software. Thus DNA microarrays using Metabolic Design can be used to perform semi-quantitative monitoring of gene expression.

### Characterization of potential metabolic capacities in a PAH contaminated soil

As we developed explorative probes to detect key genes coding for enzymes involved in PAH degradation, we assess the metabolic capacities of endogenous microbial communities in a polluted ecosystem. Owing to the difficulty in extracting microbial RNA in such environments, we hybridize total extracted microbial DNA from a highly contaminated soil (contamination details in Additional file 2). This ecosystem is selected because it harbors high concentrations of PAHs (2,300 mg/kg of dry soil). Also, PHE and FLA are detected as major contaminants (respectively 430 and 270 mg/kg of dry soil).

**Figure 4:**
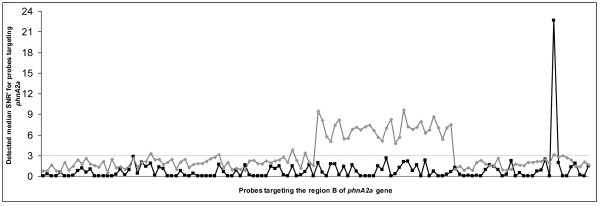
**Median SNR' for the contaminated soil with 128 specific probes targeting the *phnA2a *gene**. This graphic represents the detected median SNR' for each specific probe (ordered by sequence) derived from the degenerate defined probe **phnA2a_MD_B **targeting one particular region of *phnA2a *gene. Black squares: signals obtained with the model strain EPA505 with a mix of both pollutants (the highest signal is given by the specific probe targeting the strain EPA505 specific gene). Gray diamonds: signals obtained with total DNA extracted from the soil S3 (clearly showing a particular probe signature). The dotted line represents the defined threshold for SNR' values.

Among the 8,048 designed probes targeting the eight genes, 358 give positive signals (SNR' > 3) after hybridization with total DNA (Table 4). For each gene, probe sets show strong signals, but with variable intensities, identifying the most probable target sequence. To evaluate the explorative capacities of our probes, we first focus on the *phnA2a *gene. We compare the signal intensities between mRNA hybridization of strain EPA505 and the DNA extract from the polluted soil (Figure 4). We clearly identify the probe signature for strain EPA505 and a specific probe signature for the polluted soil. Using a BLASTn approach with complete databases (EMBL), 21 positive probe sequences have high similarities (0, 1 or 2 mismatches) with *phnA2a *genes from known PAH degraders (such as *Novosphingobium *sp. H25, *Cycloclasticus *sp. NY93E or *Sphingomonas *sp. CHY-1) (data not shown). We can then hypothesize that other positive probe sequences presenting a slight homology with available *phnA2a *sequences might have targeted *phnA2a *unknown genes, consistent with the explorative purpose of these probes.

**Table 4:** Results obtained with designed probes with total DNA extracted from the contaminated soil S3.

Gene name	*phnA1a*	*phnA2a*	*ahdA1c*	*ahdA2c*		*bphB*	*bphC*	*bphA3*	*ahdA4*
**Targeted region**	**A**	**B**	**B**	**A**	**B**	**A**	**B**	**B**	**A**

**Total number of ** **specific probes**	256	128	1024	1024	768	768	128	768	1024

**Number of specific ** **probes giving a ** **positive signal ** **(SNR' > 3)**	0	37	204	18	1	36	16	44	2

**Percentage of probes ** **giving a positive ** **signal (SNR' > 3)**	0	28.90	19.92	1.75	0.13	4.68	12.50	5.72	0.19

**Highest median SNR' ** **obtained for each ** **targeted region**	0 ± 0.00	9.47 ± 0.70	42.85 ± 5.83	7.05 ± 1.37	4.29 ± 1.71	6.33 ± 2.05	4.43 ± 1.31	8.84 ± 2.15	3.48 ± 0.98

For each degenerate probe defined targeting one particular region (A or B) of genes (*phnA1a, phnA2a, ahdA1c, ahdA2c, bphB, bphC, bphA3 *and *ahdA4*), total number of specific probes stemming from the degenerate sequence, total number of specific probes giving a 'positive' signal (with a SNR' > 3), probe percentage giving a 'positive' signal and highest signal median SNR' visualized for each targeted region of each gene.

The highest SNR' signal is given for a probe targeting *ahdA1c *(42.85 

 ±

  5.83) among 204 other positive probes for this gene. As for *phnA2a *positive probes, several are potentially explorative. Interestingly, specific probe targeting *ahdA1c *gene from strain EPA505 also gives a positive signal (median SNR' = 7.45 

 ±

  0.34). The same positive results are obtained with probes specific to strain EPA505 genes: 3.12 

 ±

  1.00 for *phnA2a*, 4.07 

 ±

  0.27 for *ahdA2c*, 4.33 

 ±

  1.14 for *bphC *and 7.06 

 ±

  1.22 for *bphA3*, suggesting the presence of bacteria closely related to strain EPA505.

Surprisingly, no probe can detect *phnA1a *gene in the polluted soil. We choose to amplify, with a PCR approach, *phnA1a *genes using degenerate primers (data not shown). The PCR products are then cloned, and eight clones are sequenced. Among these eight sequences, seven showing high similarities with *phnA1a *genes are then compared with our probe sequences. This comparison reveals multiple mismatches (data not shown), impeding hybridizations with our probes. This result indicates a marked divergence of this gene family. Our first design focused on *phnA1a *genes related to *Sphingomonas*. For a broader discovery of gene diversity, we will need to design probes that take into account more exhaustively the most complete sequence diversity in databases (international and/or personal).

## Discussion

We have developed and validated a new algorithm named Metabolic Design. This software can be used to design efficient explorative probes for functional DNA microarrays. Previously to probe design, users have to extract from public (Swiss-Prot and TrEMBL) or personal databases, protein sequences of interest. Results are then integrated in a user-friendly, intuitive interface. All databases used for the application can be selected by the users and they can also integrate personal data. Such flexibility is generally not available, for example with current metabolic reconstruction tools, such as the 'Pathway Tools Software', initially developed for the EcoCyc project [36], or KEGGanim [37]. These are generally based on static databases and predefined metabolic pathways (such as KEGG [38], MetaCyc [39] or BRENDA [40]).

In order to bypass the faulty annotations found in automatically filled databases, and to allow the exhaustive exploitation of all the currently available protein sequences, the mining step is performed using similarity search. However, such approach presents another major drawback. Indeed, in some cases, not all proteins with a similar function have similar primary structures. Thus a future development of Metabolic Design will be the replacement of the BLASTp step by a Pattern Hit Initiated BLAST (or PHI-BLAST) step coupled with PRODOM data (defined as a comprehensive set of protein domain families automatically generated from the UniProt Knowledge Database) [41]. PHI-BLAST analysis is useful for identifying the distant members of a protein family, whose relationship is not recognizable by straight sequence comparison, but only by patterns contained in sequences (such as catalytic sites or substrate recognition sites). We also intend to integrate a new module for high-throughput ortholog prediction (using for example Ortho-MCL or Ortholuge) to improve homologous protein selection for complex and divergent protein families [42,43].

The ultimate aim of Metabolic Design is to define explorative probes and estimate their specificity *in silico*. Specific probes deduced from defined degenerate probes thus allow the targeting not only of known gene sequences but also of new ones that encode the same protein sequences. These explorative features are not offered by other tools such as OligoArray 2.0, YODA or HPD [17]. In addition, Metabolic Design takes into consideration both *ex situ *and *in situ *DNA microarray synthesis. The inosine composition is taken into account in the total degeneracy, as an *ex situ *microarray can hold inosine nucleotide probes, and/or degenerate probes in one spot, reducing probe degeneracy.

Probe specificity is then evaluated *in silico *using a proprietary database, giving us a close glimpse of potential cross-hybridizations found in complex environments. In addition, in Metabolic Design, this database can be modified to consider complete DNA data, or only fragmented data (for example, only one genome). Estimation and validation of potential cross-hybridizations are performed by a BLASTn analysis. However, one possible improvement would be to take into account optimized BLASTn parameters recently described as allowing a more efficient detection of potential cross-hybridizations [44].

Another update of Metabolic Design will add thermodynamic calculations to improve probe selection, although these parameters are not fully described at present [21,45]. Also, it will be essential to take into account probe sensitivity due to sequence nature [46]. In view of these difficulties in precisely predicting probe behavior during DNA microarray hybridizations, we suggest that users first validate the quality of the DNA microarrays (probe specificity and sensitivity), with a simple biological model as we did in this study.

Based on Metabolic Design defined probes, targeting eight genes coding for enzymes involved in the degradation of various PAHs by strain EPA505, we demonstrate that our design strategy is useful for most of the determined probes. Furthermore, these results highlight the capacity of our probes for semi-quantitative monitoring of gene expression or gene detection, confirming the quantitative capability of our microarrays for environmental applications [14]. Finally, we demonstrate the explorative ability of our probes, studying a complex environment. Indeed, most classical functional microarrays (such as GeoChip) using specific probes will monitor only known sequences and cannot appraise the complete microbial gene diversity of complex environments [13,14,47,48]. Additionally, considering the high complexity of environmental samples, it will be interesting to improve again probes specificity and sensitivity, using for example the 'GoArrays' strategy [29].

To allow the identification of complete sequences of targeted genes, a further application of these explorative DNA microarrays will be the capture of 'unknown' sequences for further next-generation sequencing [49,50]. Some new techniques have been reported for performing selective capture of sequence fragments from complex mixtures based on hybridization to DNA microarrays. Combining our explorative DNA microarrays and next-generation sequencing will, for example, bypass a critical bottleneck in microbial ecology, namely the difficulty of specifically exploring some biochemical pathways or specific biomarkers without the need to sequence the complete metagenome or PCR products (not reflecting reality due to PCR artifacts). Most often in complex environments even with high throughput sequencing, we obtain only a partial view of the extremely broad microbial diversity. In addition, using mRNA or large DNA fragments as targets can allow all the genes included in a transcriptional unit to be captured. So, in prokaryotes, like genes involved in the same biological process are generally associated in the same transcriptional unit, this capture would allow to assign of new gene functions.

## Conclusions

This study evaluates the efficiency of a new probe design software tool, Metabolic Design, dedicated to DNA functional microarrays. This software, which can be used to study any group of genes, was successfully applied to define probes able to detect with high specificity and sensitivity genes encoding enzymes involved in PAH degradation. In addition, DNA microarray experiments performed on soil polluted by organic pollutants, without prior sequence assumptions, demonstrate explorative abilities of our probes. So, probe design performed with Metabolic Design ensures to precisely monitor metabolic regulations during various processes in complex environments.

## Methods

### Software implementation

The Metabolic Design application can be obtained on request *via *FTP and runs only on MS-WINDOWS (32-bit) platforms. The Java runtime environment (JRE) Version 1.4 or higher, Perl Version 1.5 or higher and an SQL database such as Oracle 9i must be installed. Latest Swiss-Prot and TrEMBL database versions have also to be downloaded for local installation of data from ftp://ftp.ebi.ac.uk/pub/databases/uniprot/current_release/knowledgebase/complete. Metabolic Design is a stand-alone multilayered tool comprising a relational database, a data object layer and a graphical user interface (GUI). For the data mining step, curated information associated with enzymes (systematic name and source organism) come from the international protein database Swiss-Prot/TrEMBL and/or personal data. Swiss-Prot/TrEMBL data are parsed using PERL scripts allowing extraction of files including sequences from prokaryotes and fungi. To evaluate potential cross-hybridization of candidate probes, microbial related sequences from the EMBL database, which include environmental samples (ENV), fungi (FUN) and prokaryote (PRO) taxonomic divisions, are selected. DNA sequences corresponding to CDSs with their respective putative 5' and 3' UTR flanking regions (arbitrarily set at 100 nt each) are then extracted and formatted to perform the cross-hybridization checking step by a tBLASTn approach ('cross-hybridization database'). For particular BLAST steps, some parameters are defined for the Metabolic Design program: tBLASTn for the specificity step (*-e 10000000 -w 2 -b 5000 -v 5000*), BLASTx for the comparison with the reference protein sequence (*-e 1e^-10 ^-F F*), BLASTn for Kane's criteria evaluation (*-e 10 -w 7 -F F -q -1*), and BLASTn for cross-hybridization clustering (*-e 10e^-10 ^-w 7*).

A JAVA classes package is developed to implement the data object layer and GUI. Object persistence is guaranteed at both text file and SQL levels.

### Chemicals

PHE, FLA, Tween 80, HPLC grade solvents and acetone are purchased from Sigma-Aldrich (Saint-Quentin-Fallavier, France). For degradation experiments, stock solutions of each PAH (PHE and FLA) are prepared in acetone at a final concentration of 2 g/L and sterilized as described above. A mixture of PHE and FLA (1 g/L each) is prepared in the same way.

### DNA extraction from soil

Total DNA is extracted from 5 g of contaminated soil (S3) following the protocol described by Zhou [51]. Three extracts are made and pooled to minimize potential biases. DNA quality is checked on a 0.8% agarose gel.

### Bacteria, growth conditions and kinetic experiments

Strain EPA505 (DSM7526) is purchased from DSMZ (Braunschweig, Germany). Cells are first grown overnight at 37°C on a shaker table (150 rpm) in 70 mL of Luria-Bertani medium (LB) containing streptomycin (100 mg/L) to produce biomass. They are then centrifuged at room temperature for 2 min at 5,000 *g *and transferred to a sterile minimum mineral medium 457 containing, for 1 L of distilled water, 2,440 mg of Na_2_HPO_4_, 1,520 mg of KH_2_PO_4_, 500 mg of (NH_4_)_2_SO_4_, 200 mg of MgSO_4 _- 7H_2_O, 50 mg of CaCl_2 _- 2H_2_O, 200 mg of Tween 80 and 10 mL of SL-4 solution as described by DSMZ [30,33]. Cultures are prepared as follows: 2 mL of the PAH stock solutions is evaporated in sterile 250 mL conical flasks, 100 mL of sterile medium 457 is added, and the flasks are inoculated with 7.5 × 10^7 ^cells prepared as above. These cultures are incubated at 28°C on a shaker table (150 rpm) for 27 h and bacterial growth is monitored spectrophotometrically at 620 nm using an Ultraspec 2000 spectrophotometer (Pharmacia Biotech AB, Uppsala, Sweden). A culture is also grown with glucose (15 g/L) as sole carbon source and energy to define the basal expression of genes implicated in PAH degradation.

### RNA extraction from strain EPA505

Total RNA from a pure culture of strain EPA505 is extracted at different times of the PAH degradation kinetics (0, 3, 6, 10, and 21 h) with the RNeasy Mini kit (Qiagen GmbH), and treated with 1.5 U of DNase I (Invitrogen) to eliminate DNA contamination. RNA sample concentration and purity are then estimated using a Nanodrop spectrophotometer (Nanodrop).

### Microarray experiments

Samples of 15 μL of strain EPA505 total RNA of four PAH degradation kinetics data points (0, 3, 6, 10 and 21 h) are enriched using the MICROB*Express*™ Bacterial mRNA Enrichment Kit (Ambion) as recommended by the suppliers. Each enriched mRNA is then amplified using the MessageAmp™ II-Bacteria RNA Amplification Kit (Ambion) with a modified protocol for the *in vitro *transcription step. Briefly, the purified double-stranded template (~14 μL) is transcribed *in vitro *with 12 μL of ATP, CTP and GTP mix (25 mM each) (Ambion), 3 μL of UTP (75 mM) (Ambion), 3 μL of amino-allyl-UTP (50 mM) (Ambion), 4 μL of 10× reaction buffer (Ambion) and 4 μL of T7 enzyme mix (Ambion) at 37°C for a 14 h incubation period. Finally, the aRNA is purified using the MessageAmp™ II-Bacteria RNA Amplification Kit (Ambion) following the manufacturer's instructions.

In the next step, 10 μg of purified aRNA for each sample are vacuum-dried and labeled using the Amersham CyDye™Post-Labeling Reactive Dye Packs (GE Healthcare, Little Chalfont, United Kingdom) with Cyanine3 or Cyanine5 dyes as recommended by the supplier. Briefly, the aRNA pellet is resuspended in 20 μL of 0.1 M bicarbonate buffer (pH 8.7) and incubated for 90 min with 40 nM of dye compound (coupling the dye to amino-allyl-UTP) dissolved in 20 μL of DMSO (dimethyl sulfoxide) in the dark at room temperature. Excess dye is quenched by adding 15 μl of 4 M hydroxylamine solution incubated for 15 min in the dark at room temperature. The labeled aRNA is then purified with NucleoSpin RNA Clean-Up kit (Macherey-Nagel, Düren, Germany) according to the manufacturer's instructions. After each step (total RNA enrichment, RNA amplification and aRNA labeling), the quantity and integrity of RNA are estimated using the RNA 6000 Nano kit (Agilent Technologies), the Agilent 2100 Bioanalyzer (Agilent Technologies) and the Nanodrop spectrophotometer (Nanodrop) as recommended by protocols.

Total DNA is amplified and labeled using the BioPrime^® ^Total Genomic Labeling System (Invitrogen) following the manufacturer's instructions. The quantity and quality of labeling are estimated using a Nanodrop spectrophotometer (Nanodrop) as recommended by protocols.

NimbleGen custom arrays of 8,048 probes are used (Roche NimbleGen, Madison, USA). All the probes are randomly distributed across the array to minimize spatial effects as far as possible during the hybridization step. The microarray also contains thousands of random probes (randomly defined length and sequence) which can serve to measure technical background noise. For each hybridization experiment, 3.33 μg of labeled RNA for kinetic experiments (one sample in Cy3 and another in Cy5) or 12 μg of labeled DNA (in Alexa Fluor^® ^5) for soil are mixed, vacuum-dried and resuspended in 5.6 μL of water. The hybridization mix (Roche NimbleGen) is then made according to the manufacturer's protocols. The arrays are hybridized on a 4-bay NimbleGen Hybridization System (Roche NimbleGen) at 42°C for 72 h. The arrays are washed with NimbleGen wash buffers I, II and III according to vendors' protocols and scanned using a Scanner Innoscan 900AL (Innopsys, Carbonne, France) at 2 μm resolution. Individual array images are acquired independently, adjusting the PMT gain for each image as recommended using Mapix^® ^software (Innopsys).

For each array image, raw expression data are extracted using the NimbleScan software v2.1. (Roche NimbleGen) and feature intensities are exported as .pair files. The background noise is then determined using random probes present on the microarrays (8,863 probes in our experiment) with the method described in the Additional file 3. This background noise is defined by two components: the background median intensity (Bposition) and its dispersion (Bdispersion). Finally, a modified signal-to-noise ratio termed SNR' and based on the formula of Verdik [52] is calculated as follows in order to reduce-centralize our data: SNR' = (probe signal intensity - Bposition)/Bdispersion (see Supplementary Data S1).

However, spatial effect across the array surface is a predominant within-slide experimental artifact that needs to be eliminated before any other normalization procedure [53]. Accordingly, for all array images obtained in this work, the surface is segmented into 16 sub-squares according to probe position (*X*, *Y*) indicated in the pair report. A Perl script is developed to calculate local background noise in all sub-squares and the median SNR' retrieved from the three replicates of each probe. Finally, another Perl script is implemented to summarize each replicate probe treated and determine the median value of the three replicates. 'Positive' hybridization is considered significant for probes with SNR' > 3 (value to avoid all false positives) [54]. The data discussed in this publication have been deposited in NCBI's Gene Expression Omnibus and are accessible through GEO Series accession number GSE21402: http://www.ncbi.nlm.nih.gov/geo/query/acc.cgi?token=rjmhhgoaqyqqinw&acc=GSE21402

### DNA extraction, PCR amplification and cloning

Total DNA from a pure culture of strain EPA505 is extracted by heat shocking cells [55]. All PCR reactions are carried out in 50 μL of mixtures containing 20 ng of the previous strain EPA505 DNA extract, 0.5 U of GoTaq DNA polymerase (Promega Corp., Madison, USA), Promega buffer, 1.25 mM MgCl_2_, 1 μM of each primer depending on the targeted gene (Additional files 4 and 5) and 0.5 mM of each deoxynucleotide. The reactions are performed in a iCycler thermal cycler (Biorad Laboratories, Marnes-la-Coquette, France) using an initial denaturation step consisting of 95°C for 5 min, followed by 35 cycles of 95°C for 1 min, an annealing step with temperature and time depending on primers (Additional Files 4 and 5) and an elongation step of 72°C for 1 min. A final treatment of 72°C for 7 min is then applied. The size and purity of PCR products are checked on 1.2% gel agarose. The PCR products are purified with a Qiaquick Gel Elution kit (Qiagen GmbH, Hilden, Germany), and then ligated into the pCRII-TOPO^® ^vector supplied with the TA cloning kit (Invitrogen Corp., Merelbeke, Belgium) and transformed into *E. coli *One Shot^® ^TOP10 cells (Invitrogen Corp.) following the manufacturer's instructions. White colonies are picked and grown in LB medium supplemented with kanamycin at 50 μg/ml final concentration. Plasmid template DNA is prepared by the alkaline lysis method [55]. The clone inserts are sequenced by the MWG Biotech Company (Ebersberg, Germany) using both SP6 and T7 sequencing primers. Sequence treatment and joining are performed using the pregap4 and the gap4 tools of the Staden Package Program [56]. The gene sequences are then compared with Swiss-Prot and TrEMBL databases using the BlastX program [57].

### Real-time PCR experiments

The reverse transcription reactions are carried out at 42°C for 2 h with 50 ng of total RNA using *bphC, bphA3, ahdA2c *and *ahdA1c *(0.625 μM of each primer) mix primers, and *ahdA4, phnA1a, phnA2a and bphB *mix primers respectively (see Additional file 6) in order to minimize manipulation biases. These reactions are carried out in a final volume of 20 μL with 100 U of SuperScriptIII reverse transcriptase (Invitrogen Corp.), 1 U of RNasin+ Inhibitor (Promega Corp.), 0.25 mM of dNTPs mix (Invitrogen Corp.), 0.1 M DTT (Invitrogen Corp.) and Invitrogen buffer, according to the manufacturer's instructions. Reverse transcription reactions are performed in triplicate. cDNA is then diluted ten-fold for quantitative real-time PCR assays. Reactions are carried out with the MESA Green qPCR for SYBR assays kit (Eurogentec) according to the manufacturer's instructions. All amplifications are carried out in a final volume of 20 μL containing 5 μL of sample described above or 5 μL of standard cDNA (from 4.37 × 10^7 ^copies/μL to 4.37 copies/μL, covering 8 log of dynamic range for each gene), 10 μL of 2× MESA Green qPCR for SYBR assays mixture and the corresponding primers sets described in the Additional file 6 at 0.2 μM final concentration each. The reverse transcription product in kinetic experiment samples is quantified twice. As every reverse transcription experiment is done in triplicate, six measurements are obtained for each sample. Each point on the standard curve (corresponding to serially diluted cDNA) is quantified in triplicate. PCR is carried out in the Mastercycler Realplex (Eppendorf, Le Percq, France) for 1 cycle at 95°C for 5 min followed by 40 cycles consisting of 95°C for 15 s (denaturation step) and 68°C for 45 s (annealing and elongation steps). At the end of the real-time PCR, a melting curve is defined by measurement of SYBR Green signal intensities for 20 min from 68°C to 95°C. Size of the amplified products is checked on 2.5% agarose gel. Data analysis is carried out with *realplex *software (version 1.5; Eppendorf).

### Nucleotide sequence accession numbers

The nucleotide sequences reported in this study have been deposited in the database under accession numbers: [EMBL: FM882255] (encompassing *bphC, bphA3, ahdA2c *and *ahdA1c *gene sequences), [EMBL: FM882254] (encompassing *phnA1a *and *phnA2a *gene sequences), [EMBL: FM882253] (encompassing *ahdA4 *gene sequences) and [EMBL: FN552592] (encompassing *bphB *gene sequences).

## List of abbreviations

GUI: graphical user interface; IUPAC: international union of pure and applied chemistry; PAH: polycyclic aromatic hydrocarbon; PHE: phenanthrene; FLA: fluoranthene; SNR': signal to noise ratio; ORF: open reading frame; CDS: coding DNA sequence; RT-PCR: reverse transcription- polymerase chain reaction; FTP: file transfer protocol; JRE: java runtime environment; SQL: structured query language; UTR: untranslated region, aRNA: antisense RNA; DMSO: dimethyl sulfoxide; cDNA: complementary DNA.

## Availability and Requirements

**Project name**: Metabolic Design

**Project homepage**: ftp://195.221.123.90/

**Operating system**: Windows (32-bit) only

**Programming language**: Java and Perl

**Others**: The Java runtime environment (JRE) Version 1.4 or higher, Perl Version 1.5 or higher and an SQL database such as Oracle 9i must be installed.

**License**: Free for non-commercial use. Source code available upon request.

## Authors' contributions

ST, EP, AM and OG carried out the experimentations and have participated to analysis and interpretation of data. FG, EP, ST and OG have made script developments, updates and optimizations of Metabolic Design. ST, EDB, FG, EP and OG performed *in silico *analysis. ST, EP, OG and PP planned the study and wrote the manuscript. JT, EP, OG, DB, CBP and PP have given final approval of the version to be published. All authors read and approved the final manuscript.

## Supplementary Material

Additional file 1 **SNR' profiles detected with microarray experiments and transcript numbers profiles detected with quantitative RT-PCR assays**. SNR' profiles detected with microarray experiments (LEFT), and transcript copy number detected per ng of total RNA with quantitative RT-PCR assays (RIGHT) for eight genes: (A) *phnA1a*; (B) *phnA2a*; (C) *ahdA1c*; (D) *ahdA2c*; (E) *bphB*; (F) *bphC*; (G) *bphA3*; and ahdA4 (H) during PAH biodegradation at different times with strain EPA505. PHE: grey squares, FLA: triangles, PHE + FLA: circles, glucose: open diamond. Error bars indicate the standard deviation of measures. Click here for file

Additional file 2 **PAH composition detected in the contaminated soil S3**. These data are proprietary data given by BioBasic Environnement and give the quantity of detected PAHs in mg/kg of dry soil in the contaminated soil studied. Click here for file

Additional file 3 **Background noise calculation description**. Background noise is determined according to 'RANDOM probes response' of Nimblegen microarrays. Our method takes into account the background noise which is characterized by two components: its position and its dispersion. Click here for file

Additional file 4 **Identification of four catabolic genes clusters from the model strain EPA505**. Physical maps of four clusters (A, B, C and D) of catabolic genes involved in PAHs biodegradation from strain EPA505. Size of genes and intergenic spaces is indicated as well as position of primers used for PCR amplifications. Click here for file

Additional file 5 **Primer sets used for detecting catabolic genes involved in PAHs degradation and to generate the gene DNA matrix**. The DNA matrix is used to build the standard curve for quantitative real-time PCR assays in strain EPA505. *: *xylX *and *nahD *are used to characterize complete sequences of *bphC *and *ahdA1c*. Nomenclature: **M**: A and C; **R**: A and G; **W**: A and T; **S**: G and C; **Y**: C and T; **K**: G and T; **V**: A, G and C; **H**: A, C and T; **D**: A, G and T; **B**: G, T and C; **I**: A, C, G and T. Click here for file

Additional file 6 **Primers used for reverse transcription and quantitative real-time PCR assays**. List of primers used for reverse transcription and subsequent quantitative real-time PCR assays. Amplification sizes are also given for each targeted gene. Click here for file
