# Supplementary material for: Detecting variants with Metabolic Design, a new software tool to design probes for explorative functional DNA microarray development
Source: BMC Bioinformatics. 2010 Sep 23;11:478. doi: 10.1186/1471-2105-11-478 (PMC2955052; doi:10.1186/1471-2105-11-478)
Supplement: Additional file 1 — SNR' profiles detected with microarray experiments and transcript numbers profiles detected with quantitative RT-PCR assays. SNR' profiles detected with microarray experiments (LEFT), and transcript copy number detected per ng of total RNA with quantitative RT-PCR assays (RIGHT) for eight genes: (A) phnA1a; (B) phnA2a; (C) ahdA1c; (D) ahdA2c; (E) bphB; (F) bphC; (G) bphA3; and ahdA4 (H) during PAH biodegradation at different times with strain EPA505. PHE: grey squares, FLA: triangles, PHE + FLA: circles, glucose: open diamond. Error bars indicate the standard deviation of measures. [file 1471-2105-11-478-S1.DOC]

| Gene Name | DNA Microarray assays | Quantitative RT-PCR assays |
| --- | --- | --- |
| (A) *phnA1a* |  |  |
| (B) *phnA2a* |  |  |
| (C) *ahdA1c* |  |  |
| (D) *ahdA2c* |  |  |
| (E) *bphB* |  |  |
| (F) *bphC* |  |  |
| (G) *bphA3* |  |  |
| (H) *ahdA4* |  |  |
