# Supplementary material for: Detecting variants with Metabolic Design, a new software tool to design probes for explorative functional DNA microarray development
Source: BMC Bioinformatics. 2010 Sep 23;11:478. doi: 10.1186/1471-2105-11-478 (PMC2955052; doi:10.1186/1471-2105-11-478)
Supplement: Additional file 2 — PAH composition detected in the contaminated soil S3. These data are proprietary data given by BioBasic Environnement and give the quantity of detected PAHs in mg/kg of dry soil in the contaminated soil studied. [file 1471-2105-11-478-S2.DOC]

| **Total PAH detected in S3**  **(mg / kg of dry soil)** | |
| --- | --- |
| Naphthalene | 620 |
| Acenaphthylene | 110 |
| Acenaphthene | 14 |
| Fluorene | 47 |
| Phenanthrene | 430 |
| Anthracene | 160 |
| Fluoranthene* | 270 |
| Pyrene | 210 |
| Benzo(a)anthracene | 79 |
| Chrysene | 74 |
| Benzo(b)fluoranthene*° | 93 |
| Benzo(k)fluoranthene*° | 36 |
| Benzo(a)pyrene* | 70 |
| Dibenzo(ah)anthracene | < 9 |
| Indenol(1,2,3-cd)pyrene*° | 37 |
| Benzo(ghi)perylene*° | 43 |
| **TOTAL** | **2,300** |
| **TOTAL (*)** | **549** |
| **TOTAL (°)** | **209** |
