# Supplementary material for: Detecting variants with Metabolic Design, a new software tool to design probes for explorative functional DNA microarray development
Source: BMC Bioinformatics. 2010 Sep 23;11:478. doi: 10.1186/1471-2105-11-478 (PMC2955052; doi:10.1186/1471-2105-11-478)
Supplement: Additional file 3 — Background noise calculation description. Background noise is determined according to 'RANDOM probes response' of Nimblegen microarrays. Our method takes into account the background noise which is characterized by two components: its position and its dispersion. [file 1471-2105-11-478-S3.DOC]

Background noise was determined according to ‘RANDOM probes response’ of Nimblegen microarrays. These probes are random oligonucleotides sequence which are randomly positioned across the microarray surface and can serve as a metric of the background noise (non-specific annealing and background fluorescence). Our method takes into account the background (B) noise which is characterized by two components: its position (Bposition) (1) and its dispersion (Bdispersion) (2):

(1) Bposition is the median intensity of all RANDOM probes considered (in the entire image or in a sub-square).

(2) Bdispersion represents the variation observed across RANDOM probes intensity values. Bdispersion is more difficult to apprehend than Bposition since it greatly depends on ‘image cleanness’.

The ‘image cleanness’ is determined and the Bdispersion is calculated according to the variation coefficient (V) observed on all RANDOM probes considered:

- If V>33.33%: image is considered as ‘dirty’ and (Bdispersion = Bposition - Q3) where Q3 is the third quartile calculated from all RANDOM probes intensities considered.

- If V<33.33%: image is considered as ‘clean’ and (Bdispersion = Bposition - D8) where D8 is the eighth decile calculated from all RANDOM probes intensities considered.
