# Supplementary figures and images for: Detecting variants with Metabolic Design, a new software tool to design probes for explorative functional DNA microarray development
Source: BMC Bioinformatics. 2010 Sep 23;11:478. doi: 10.1186/1471-2105-11-478 (PMC2955052; doi:10.1186/1471-2105-11-478)

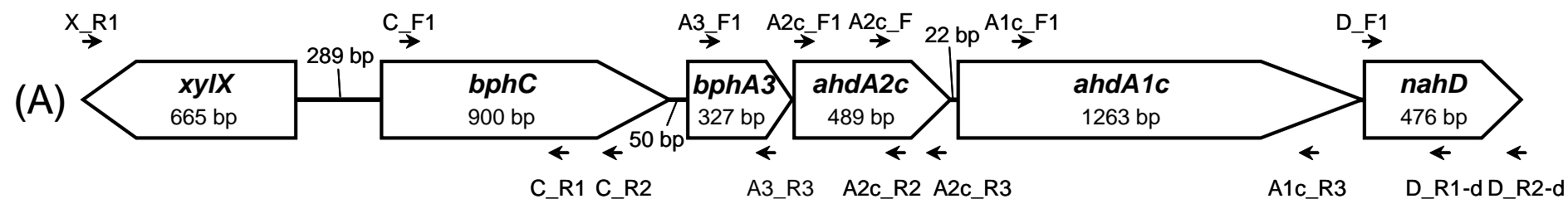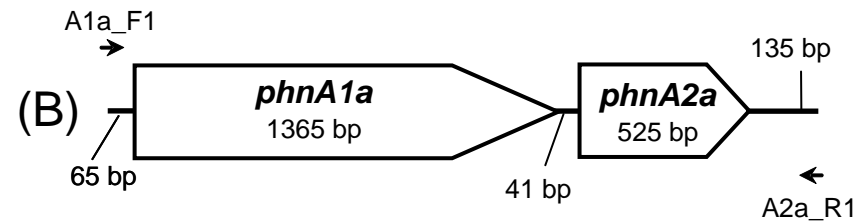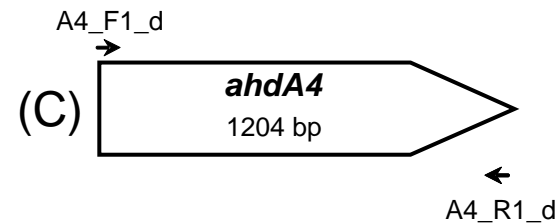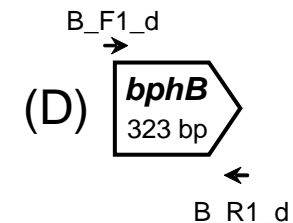

Supplement: Additional file 4 — Identification of four catabolic genes clusters from the model strain EPA505. Physical maps of four clusters (A, B, C and D) of catabolic genes involved in PAHs biodegradation from strain EPA505. Size of genes and intergenic spaces is indicated as well as position of primers used for PCR amplifications. [file 1471-2105-11-478-S4.PDF]
