# Supplementary material for: Detecting variants with Metabolic Design, a new software tool to design probes for explorative functional DNA microarray development
Source: BMC Bioinformatics. 2010 Sep 23;11:478. doi: 10.1186/1471-2105-11-478 (PMC2955052; doi:10.1186/1471-2105-11-478)
Supplement: Additional file 5 — Primer sets used for detecting catabolic genes involved in PAHs degradation and to generate the gene DNA matrix. The DNA matrix is used to build the standard curve for quantitative real-time PCR assays in strain EPA505. *: xylX and nahD are used to characterize complete sequences of bphC and ahdA1c. Nomenclature: M: A and C; R: A and G; W: A and T; S: G and C; Y: C and T; K: G and T; V: A, G and C; H: A, C and T; D: A, G and T; B: G, T and C; I: A, C, G and T. [file 1471-2105-11-478-S5.DOC]

| **Gene fragment** | **Forward primer** | **Sequence (5’ – 3’)** | **Reverse primer** | **Sequence (5’ – 3’)** | **Annealing**  **Temp (°C)** | **Annealing time (sec)** |
| --- | --- | --- | --- | --- | --- | --- |
| Primers used to detect catabolic genes | | | | | | |
| *xylX* – bphC* | X_R1 | ACCTGCASCTTCCAGTTGCC | C_R1_d | CKYTCRTTRCARTGCATRAA | 45 | 45 |
| *bphC* | C_F1_d | GAYYTBTGGCAYCAYCGCAT | C_R1_d | CKYTCRTTRCARTGCATRAA | 45 | 30 |
| *bphC – bphA3* | C_F1_d | GAYYTBTGGCAYCAYCGCAT | A3_R3_d | TGRCAIGGRAAIGCYTT | 40 | 30 |
| *bphA3* | A3_F1_d | ACIGAYGGITAYCARGAY | A3_R3_d | TGRCAIGGRAAIGCYTT | 40 | 30 |
| *bphA3 – ahdA2c* | A3_F1_d | ACIGAYGGITAYCARGAY | A2c_R2_d | TSRTCIAYRTAYTTICC | 40 | 30 |
| *ahdA2c* | A2c_F1_d | GAYGAYGAYMGIYTIGAR | A2c_R3_d | ACCATCATIGTRTCDAT | 40 | 30 |
| *ahdA2c – ahdA1c* | A2c_F | CCTTATGACCCGCACTGACG | A1c_R3_d | GCYTCISYRTCYTCCAT | 53 | 30 |
| *ahdA1c* | A1c_F1_d | TGYGTITAYCAYCARTGG | A1c_R3_d | GCYTCISYRTCYTCCAT | 40 | 30 |
| *ahdA1c – nahD** | A1c_F1_d | TGYGTITAYCAYCARTGG | D_R2_d | TBCGIGCCTTGCGRTATTC | 40 | 30 |
| *phnA1a – phnA2a* | A1a_F1 | CATCGCATTGCCATTAGTG | A2a_R1_d | CTTGACKAGYACCTCRCCRT | 56 | 30 |
| *ahdA4* | A4_F1_d | GWGCRAATCTKGCSGGTGG | A4_R1_d | ARCCMGCCTGCTTGAGSA | 56 | 30 |
| *bphB* | B_F1_d | TTYGGIAARYTBGAYGT | B_R1_d | GGIGCVACICCRTTVAC | 50 | 40 |
| Primers used to generate gene DNA matrix for quantitative reverse transcription PCR assays | | | | | | |
| *bphC* | C_F1 | TGGGAGAGAAAGCAAATGG | C_R1 | TAATGGAAGGCTCAACCGA | 56 | 30 |
| *bphA3* | A3_F1 | GCTGACCTTCTACTGCGCCA | A3_R1 | CGGTGTAGACGCAGTCCGAA | 68 | 15 |
| *ahdA2c* | A2c_F1 | GGTTCCTTCGACATCGCCAC | A2c_R1 | AACGGCGATCTTTGAGCGGA | 68 | 15 |
| *ahdA1c* | A1c_F1 | GGACGCACCACAATCTACAAT | A1c_R1 | TATCTTGCGGGTCATCGTG | 60 | 30 |
| *phnA1a* | A1a_F1 | CATCGCATTGCCATTAGTG | A1a_R1 | GGCGTCACCGGAACTTGTTC | 56 | 30 |
| *phnA2a* | A2a_F1 | CAGAGCCGGTCCAAATATCG | A2a_R1 | CTTGACTAGTACCTCGCCGT | 59 | 30 |
| *ahdA4* | A4_F1_d | GWGCRAATCTKGCSGGTGG | A4_R1_d | ARCCMGCCTGCTTGAGSA | 56 | 30 |
| *bphB* | B_F1_d | TTYGGIAARYTBGAYGT | B_R1_d | GGIGCVACICCRTTVAC | 50 | 40 |
