# Supplementary material for: Detecting variants with Metabolic Design, a new software tool to design probes for explorative functional DNA microarray development
Source: BMC Bioinformatics. 2010 Sep 23;11:478. doi: 10.1186/1471-2105-11-478 (PMC2955052; doi:10.1186/1471-2105-11-478)
Supplement: Additional file 6 — Primers used for reverse transcription and quantitative real-time PCR assays. List of primers used for reverse transcription and subsequent quantitative real-time PCR assays. Amplification sizes are also given for each targeted gene. [file 1471-2105-11-478-S6.DOC]

| *Gene* | *Reverse transcription* | *Quantitative real-time PCR* | | |
| --- | --- | --- | --- | --- |
|  | primer | forward primer | reverse primer | Fragment size (bp) |
| *bphC* | AAGCCCGAAAGCGACCGAAT | TTTTACGGGCCGCAAGTCGA | GGAACATCGTCCTGACGCAG | 120 |
| *bphA3* | CCTTCCGGCTGATCGATGCA | GCCATGCTGACCGATGGCTA | GTGGCGATGTCGAAGGAACC | 77 |
| *ahdA2c* | CGTCAGTGCGGGTCATAAGG | CTGCAGCAACTCGTGACCGA | CGGTGTAGACGCAGTCCGAA | 106 |
| *ahdA1c* | ATGGCGATGAAGGAGGTTGC | CCCTATCACGCCAGCCTTCT | AATGCCGCTTCTCGCTATCC | 100 |
| *phnA1a* | GACCGGCACTTTCCAGTTGC | CAGATTTGCCACGCCGACAG | TTCGGGATCATGGCAACCGA | 204 |
| *phnA2a* | CGTGACTCTCGCATCGAGGA | GTCGAAGCCTTTGAAGCCGA | TGAAACCATTGCCGTCCTGG | 139 |
| *ahdA4* | TGTTGACCTCGATCCCGGCA | CCAGTGCCGATGGAAAGAGC | CAAACCTGAAGCCGGTCACG | 115 |
| *bphB* | TCGATGCGACATACAGCGTC | GACGTCAACCTGAAGGGGTA | TCGATGCGACATACAGCGTC | 139 |
